# Searching Embase and MEDLINE by using only major descriptors or title and abstract fields: a prospective exploratory study

**DOI:** 10.1186/s13643-018-0864-9

**Published:** 2018-11-20

**Authors:** Wichor M. Bramer, Dean Giustini, Jos Kleijnen, Oscar H. Franco

**Affiliations:** 1 Medical Library, Erasmus MC, University Medical Centre Rotterdam, P.O. Box 2040, 3000 CS Rotterdam, The Netherlands; 2 UBC Biomedical Branch Library, The University of British Columbia, Vancouver, Canada; 3 Kleijnen Systematic Reviews Ltd, York, UK; 4 School for Public Health and Primary Care (CAPHRI), Maastricht University, Maastricht, The Netherlands; 5 Department of Epidemiology, Erasmus MC, University Medical Centre Rotterdam, Rotterdam, The Netherlands; 6 Institute of Social and Preventive Medicine, University of Bern, Bern, Switzerland

**Keywords:** Databases, Bibliographic, Review literature as topic, Sensitivity and specificity, Information storage and retrieval

## Abstract

**Background:**

Researchers performing systematic reviews (SRs) must carefully consider the relevance of thousands of citations retrieved from bibliographic database searches, the majority of which will be excluded later on close inspection. Well-developed bibliographic searches are generally created with thesaurus or index terms in combination with keywords found in the title and/or abstract fields of citation records. Records in the bibliographic database Embase contain many more thesaurus terms than MEDLINE. Here, we aim to examine how limiting searches to major thesaurus terms (in MEDLINE called focus terms) in Embase and MEDLINE as well as limiting to words in the title and abstract fields of those databases affects the overall recall of SR searches.

**Methods:**

To examine the impact of using search techniques aimed at higher precision, we analyzed previously completed SRs and focused our original searches to major thesaurus terms or terms in title and/or abstract only in Embase.com or in Embase.com and MEDLINE (Ovid) combined. We examined the total number of search results in both Embase and MEDLINE and checked whether included references were retrieved by these more focused approaches.

**Results:**

For 73 SRs, we limited Embase searches to major terms only while keeping the search in MEDLINE and other databases such as Web of Science as they were. The overall search yield (or total number of search results) was reduced by 8%. Six reviews (9%) lost more than 5% of the relevant references. Limiting Embase and MEDLINE to major thesaurus terms, the number of references was 13% lower. For 15% of the reviews, the loss of relevant references was more than 5%. Searching Embase for title and abstract caused a loss of more than 5% in 16 reviews (22%), while limiting Embase and MEDLINE that way this happened in 24 reviews (33%).

**Conclusions:**

Of the four search options, two options substantially reduced the overall search yield. However, this also resulted in a greater chance of losing relevant references, even though many references were still found in other databases such as Web of Science.

**Electronic supplementary material:**

The online version of this article (10.1186/s13643-018-0864-9) contains supplementary material, which is available to authorized users.

## Background

Performing high-quality systematic reviews (SRs) is an exacting and time-consuming process because biomedical researchers are required to review thousands of titles and abstracts from their searches in traditional bibliographic databases. Ultimately, the percentage of references selected for inclusion in a SR is around 2% [1]. The time and resources needed for selection and screening of papers can be considerably reduced by reducing the number of papers found by a more targeted search. Still, targeted searching and its benefits should be evaluated against the likelihood of missed relevant studies.

Many peer-reviewed articles are retrieved by searching bibliographic databases such as MEDLINE and Embase [2–4]. Including terms from a *controlled vocabulary*, or thesaurus such as Medical Subject Headings (MeSH) in MEDLINE and Excerpta Medica Thesaurus (Emtree terms) in Embase is critical in creating robust, sensitive searches. “A controlled vocabulary is an organized arrangement of words and phrases used to index content and/or to retrieve content through browsing or searching. It typically includes preferred and variant terms and has a defined scope or describes a specific domain.” [5]. In the databases Embase and MEDLINE, either indexers read articles and select predefined terms that closely describe their content, or this is done by an automated algorithm. A few of the most important assigned thesaurus terms (usually around 25%) are marked as major descriptors or focus concepts [6]. There are many ways to describe the phenomenon (such as major topic, major terms, focus terms, major headers); for clarity, we will use the term major descriptor throughout the article for both databases.

When conducting SRs, searches are designed to be more sensitive in order to retrieve as many relevant papers as possible. Combining thesaurus terms searched in subject fields with words or key terms in the title and abstract fields are recommended for good recall [7].

Embase records generally have more thesaurus terms attached to them than records in MEDLINE. Thus, searching with Embase (Emtree) terms can increase the likelihood of finding more references that might not necessarily be relevant [8–10]. The number of search results retrieved by a search strategy can be reduced by searching for index terms as major descriptors or by searching title and abstract fields only [11]. It is unclear how focusing search strategies affects the total number of search results for SRs, and whether potentially relevant references would be missed.

Previous research on focusing search strategies is not clear about the impact on recall when using or targeted field search strategies [12, 13]. In the existing literature, overall performance of searches is determined by examining the final set of relevant references. Data for individual SRs suggests that there are many differences in the performance of searches aimed at a higher precision. However, only Embase is usually taken into account [13]. Still, in biomedical SRs, any articles missed in Embase may be found by other databases such as MEDLINE or Web of Science.

We aimed to determine whether focused Embase and MEDLINE searches negatively impacted the retrieval of relevant studies across a broad range of SRs.

## Methods

This cross-sectional study is reported along the recommendation of the STROBE checklist (see Additional file 1). To examine more focused searches and their impact, we looked at individual reviews, rather than on overall performance combining multiple reviews. We took into account the search results from additional databases, which might find articles not retrieved in Embase and MEDLINE.

### Registration of systematic review searches

From May 2013 to present, we registered SR search strategies and documented results for researchers at Erasmus MC (Erasmus University Medical Centre, Rotterdam, the Netherlands). These data were tested in other research projects regarding the coverage and retrieval of certain bibliographic databases in supporting SRs [14]. Once these searches were performed, the results were imported into EndNote [15] and saved before and after deduplication. Deduplicated EndNote files for each SR were assessed by the researchers who requested the search in order to determine the references most relevant to their specific research question, for inclusion in their review. EndNote files that had not been deduplicated were saved for later analysis.

### Collecting Erasmus MC published reviews

We searched PubMed retrospectively for SRs published by researchers from Erasmus MC. We included published reviews where the Erasmus MC affiliation was listed for the first or corresponding author and for which we had documented the searches. In all these reviews, we searched at least in Embase.com, Ovid MEDLINE, and Web of Science and often additional databases (in particular the Cochrane Library and Google Scholar). All search strategies were designed by the same Erasmus MC librarian (Wichor Bramer), and all searches combined thesaurus terms and searches for words or phrases in titles and abstract fields in Embase and MEDLINE.

Of the SRs analyzed here, all references that were selected for inclusion were aggregated. All included references were searched one-by-one in the EndNote file containing the original downloaded search results from all databases for the related SR. By using record numbers of search results in EndNote, we determined which databases had retrieved the specific reference in question.

### Adaptation of original search strategies

The original search strategies used in Embase and MEDLINE were modified in four ways: (1) by searching Embase thesaurus terms as major descriptors, (2) by removing thesaurus terms from the Embase search such that only terms were searched in the title and/ or abstract fields, (3) by searching both MEDLINE and Embase for major thesaurus terms, and (4) by searching both MEDLINE and Embase for terms in the title and or abstract fields only. For searches where major thesaurus terms were used to limit retrieval, we searched non-major thesaurus terms for concepts related to study type and age groups such as children. These are so-called *check tags*, which are by default never marked as major terms. In the title and/or abstract only searches, we removed all thesaurus terms, including the check tags for age groups and searched those elements of the search also as title and/or abstract only. In Additional file 2: Table S1, an example of adapted search strategies is given.

### Characteristics of the included reviews

We analyzed review topics by using the MeSH tree structures in the MeSH Database (on Entrez) [16]. For the most important disease aspect, we selected the broadest or highest level MeSH term below the *Diseases Category* in the tree. For the most important intervention, we chose the MeSH term directly below the *Analytical, Diagnostic and Therapeutic Techniques and Equipment Category* tree. However, for terms designated under *Therapeutics*, we used those MeSH term at one level deeper. We also documented the domain of the review out of seven predefined domains. Reviews on the effectiveness of a treatment were designated *therapy*; policies were designated *management*. Research on incidence and prevalence were designated as *epidemiology*, and reviews on causes of diseases as *etiology*. Other domains we documented were *prognosis*, *diagnosis*, and *prevention*.

### Analysis of included references

Two sets of included references were created for each review: (1) a set with included references retrieved only by searching in Embase and (2) a set of included references that were uniquely retrieved by Embase and/or MEDLINE. Included references that had been identified by other searches (such as Web of Science, Cochrane CENTRAL, or handsearching) were unaffected as we did not change those searches. Specific references were searched in both databases by combining authors’ names with distinct title words. In Embase, we combined all references uniquely retrieved by Embase into a single set, while in MEDLINE we did the same for all references uniquely retrieved through a combination of Embase and MEDLINE. We combined each modified focused search in Embase and MEDLINE (two per database) with the set of references found in those databases for each review.

**Fig. 1:**
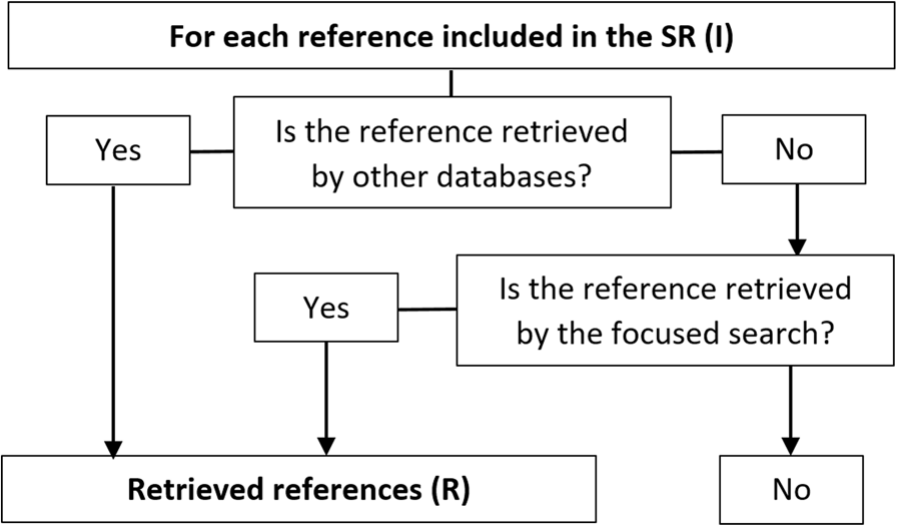
Flow chart of references included and found by focused searches

We calculated the sensitivity of these more focused searches by dividing the number of references found (sum of the total unique included references found by the focused search and total number of included references found by other databases) by the total number of references included (Fig. 1).

We could not determine the exact number of references retrieved with focused search strategies because the focused searches were created retrospectively for reviews that had been published for which the searches had been executed months or years before this research project. We therefore estimated the relative reduction in overall number of search results based on the number of unique results from both databases, combined multiplied by the ratio in reduction of number of results by examining the focused searches. For this calculation we used the numbers as indicated in Table 1.

**Table 1:** Estimation of number of search results of a focused search for a systematic review

	Before deduplication	After deduplication
Embase.com	Eb	Ea
Medline Ovid	Mb	Ma
Web of Science	Wb	Wa
Other databases	Ob	Oa
Total	Tb	Ta

Deduplication for the reviews was always done as follows: Embase records were imported first, followed by MEDLINE and then other databases. The number of records from other databases never changes. The number of unique references in Embase after deduplication (Eu) is Ea − (Mb − Ma). The number of unique references in Medline after deduplication (Mu) is Ma − (Wb − Wa). We calculated the ratio that a focused search in Embase (rE) and MEDLINE (rM) as the number retrieved with the focused search divided by the number of the original search. The total number of references after deduplication with a focused search is embase = Oa + Wa + Ma + rE*Ea, and for the focused search in MEDLINE and Embase combined Oa + Wa + rM*Ma + rE*Ea.

## Results

We collated all included references from 73 published systematic reviews for a total of 2551 references. Of these included references, 163 (6.4%) were retrieved by Embase alone, and 369 references (14.5%) were not retrieved by any other database than Embase or MEDLINE. The topics of the included reviews are described in Table 2. By limiting Embase to major thesaurus terms, the overall number of search results for individual reviews was reduced by a median of 8%. Those searches retrieved a total of 2515 references (98.6%). Of the 163 references that had been retrieved uniquely by Embase, 127 references (77.9%) were found if the searches were limited to major thesaurus terms. In 57 of 73 reviews (78%), all included references would still have been found in Embase if searches had been limited to major terms only. In the Additional file 2, an overview of the included reviews and the results is given.

**Table 2:** Description of reviews included in the research

Patient (*N* = 62)	
Wounds and injuries	7 (11%)
Cardiovascular diseases	7 (11%)
Musculoskeletal diseases	6 (10%)
Nutritional and metabolic diseases	5 (8%)
Neoplasms	5 (8%)
Pathological conditions, signs and symptoms	4 (6%)
Female urogenital diseases and pregnancy complications	4 (6%)
Intervention (*N* = 41)	
Chemicals and drugs	12 (29%)
Operative surgical procedures	12 (29%)
Domain (*N* = 68)	
Etiology	17 (25%)
Therapy (non-RCT)	13 (19%)
Therapy (RCT)	10 (15%)
Management	9 (13%)
Epidemiology	7 (10%)
Diagnosis	7 (10%)
Prognosis	5 (7%)

By limiting Embase searching to title and abstract fields, the median reduction in number of search results per review was 11%, retrieving a total of 2472 references (96.9%). Of the 163 references that were uniquely retrieved by Embase, 84 (51.1%) were retrieved by the search in title and/or abstract only. In those searches, 50 reviews (68%) were unaffected in terms of lost included articles. When we limited both Embase and MEDLINE to major thesaurus terms, median reduction in number of search results was 13%, retrieving 2487 references (97.5%). Of the 369 included references that had not been retrieved by other databases than MEDLINE or Embase, 305 (82.7%) were retrieved when both Embase and MEDLINE had been limited to major thesaurus terms only. In 46 reviews (63%), no included references were missed. Limiting both Embase and MEDLINE to title and abstract field searches reduced the number of search results by 20% and retrieved 2381 included references (93.3%). Of the 369 included references that had not been retrieved by databases beyond MEDLINE or Embase, 199 (53.9%) were retrieved when both the MEDLINE and Embase searches were limited to words in title and/or abstract only. In other words, 38 reviews (52%) would still have retrieved all included references using these more focused strategies.

**Fig. 2:**
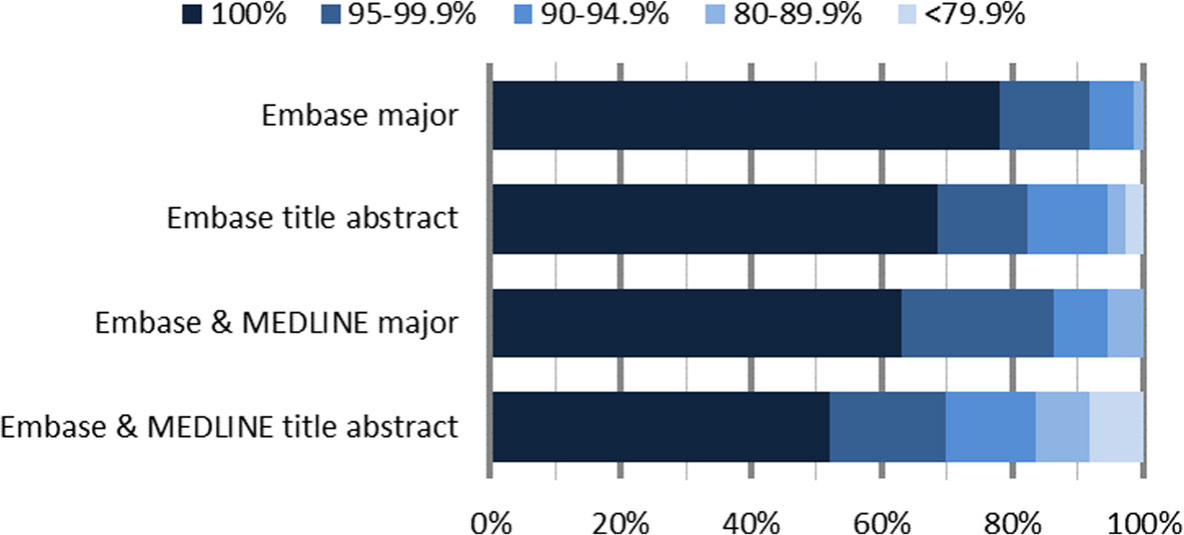
Percentage of reviews that reached a certain recall threshold using focused searches

In Fig. 2, we show the percentage of reviews for which the focused searches reached a certain recall threshold. For instance, 91% of all reviews achieved 95% recall or greater when Embase searches were limited to major Emtree index terms. Similarly, 80% of all reviews had at least 90% recall when both Embase and MEDLINE were used to search titles and/or abstracts only.

**Fig. 3:**
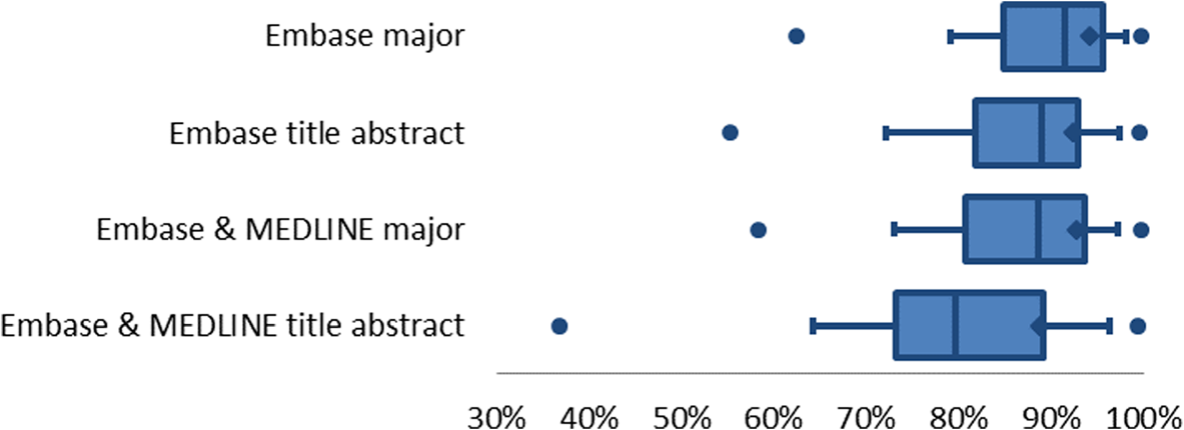
Ratio between total number of search results for systematic reviews for focused searches and the original unchanged searches. The figure shows from left to right: minimum, 10th percentile, 25th percentile, average, median, 75th percentile, 90th percentile, and maximum

Figure 3 shows the change in the total number of search results retrieved by the more focused searches. Limiting Embase to major thesaurus terms retrieved the highest number of search results, and limiting both databases to titles and abstracts retrieved the lowest. If both Embase and MEDLINE were restricted to major thesaurus terms, fewer search results were retrieved than by limiting only Embase to titles and abstracts.

**Fig. 4:**
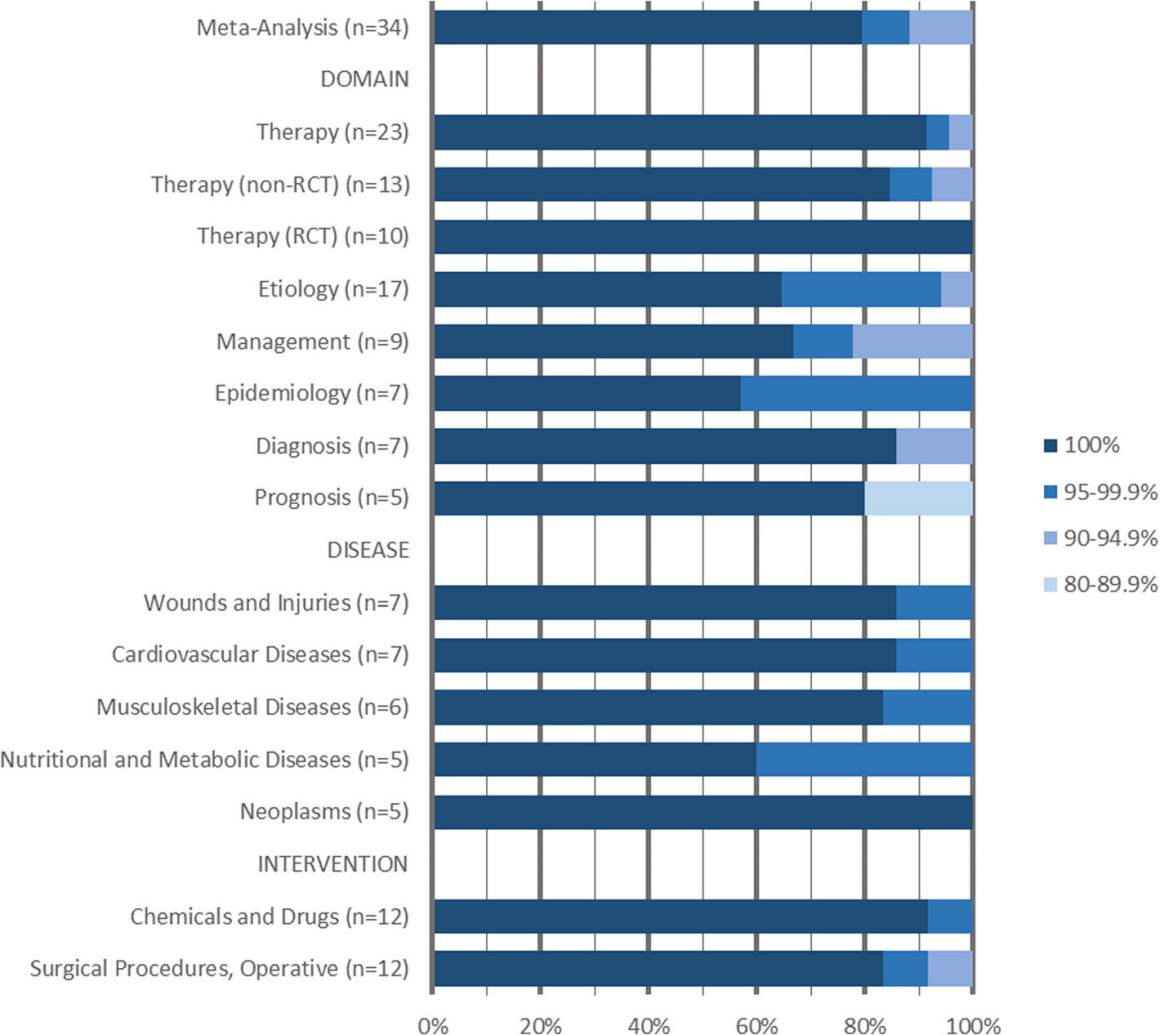
The sensitivity of searches for all databases where Embase was focused to major thesaurus terms for different review types and topics

Since limiting Embase to major descriptors resulted in the most acceptable results, we decided to investigate that search type further for different review types. For the review types of which we identified five or more reviews, we analyzed these results separately. Figure 4 shows the results of limiting Embase to major thesaurus terms only for the reviews that performed a meta-analysis, all seven domains we identified, five of the most frequently observed types of diseases and two types of interventions. For two subsets of reviews, the searches in Embase limited to major thesaurus terms retrieved all included references for all reviews. The 10 reviews in the therapeutic domain that included only randomized controlled trials and the 5 reviews about neoplasms were not affected by limiting Emtree terms to major. Here, more focused searches might still generate acceptable results (Table 2).

**Fig. 5:**
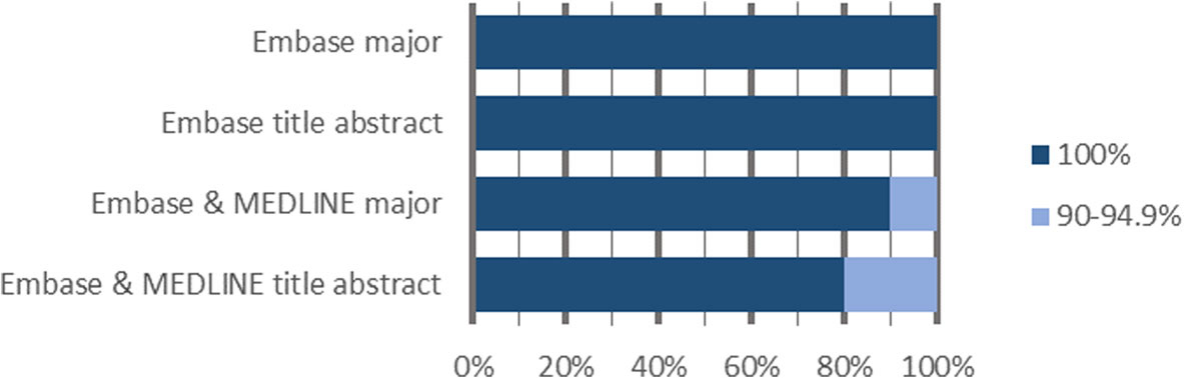
The effect of the four focused search methods on therapeutic reviews that included only RCTs (*N* = 10)

**Fig. 6:**
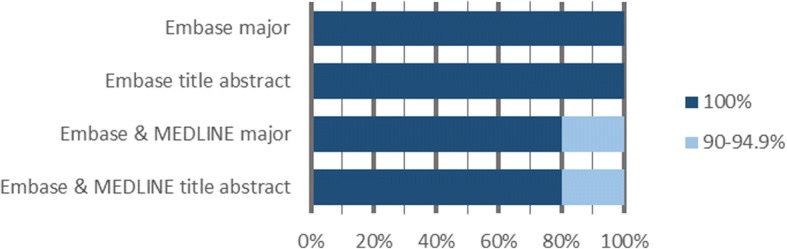
The effect of the four focused search methods on reviews about oncology (*N* = 5)

For reviews that included RCTs only, or where the main disease was cancer, we analyzed in more detail the effects of the focused searches by searching in the title and/or abstract fields only in Embase and by focusing MEDLINE searches further. Figures 5 and 6 show the results of these analyses. Both types show that focusing Embase did not negatively affect the included references. When MEDLINE was limited further, some reviews missed included references. This was because Embase did not find any unique references for the therapeutic reviews that included only randomized controlled trials (RCTs), and Embase found only one unique included reference for reviews in neoplasms.

## Discussion

In this paper, we have compared four different search methods to reduce the total yield of articles for screening. For each method, the aim was to determine the likelihood that a more focused search might miss relevant articles. The method with the fewest negative consequences (option 1: limiting searches in Embase to major thesaurus terms) only reduced the overall number of search results by 8%. Two methods that resulted in a greater reduction of search results but with acceptable recall for most SRs were (2) searching Embase for terms in the titles and abstract fields only and (3) limiting searches in both Embase and MEDLINE to major descriptors. The method that found the fewest search results is method 4: limiting Embase and MEDLINE searches to terms in titles or abstracts alone which resulted in a substantial loss of relevant papers where more than 95% of the included references in fewer than 67% of the SRs would have been retrieved.

To our knowledge, this is the first study to investigate the possible effects of using more targeted search strategies in Embase and MEDLINE. In 2015, investigators from the Canadian Agency for Drugs and Technologies in Health (CADTH) published an extensive report on the topic [12]. Still, because sensitivity scores in their original searches were low to begin with, sometimes even less than 10%, focusing them further would have been problematic. We broadened the scope of the previous research by looking at both Embase and MEDLINE and bij searching for title/abstracts only terms.

We observed that for therapeutic reviews that included RCTs only, and for reviews on neoplastic diseases, focusing the Embase searches did not have a negative effect on the set of included references. However, the number of reviews in both of these groups is small. The generalizability of this observation might not be high.

In this research, it was not necessary to confirm that all included references from all reviews were found. Here, data collected during previous research was reused and examined in different detail [14]. This previous work aimed to identify the databases from which all included references had been retrieved. For the references that had been retrieved from databases other than Embase and MEDLINE, most notably Web of Science, we did not change our search strategies. The goal was to determine the recall of the more targeted searches by examining only those references that were uniquely retrieved by Embase and/or MEDLINE. For reviews in which all included references had also been retrieved by databases other than Embase and MEDLINE, recall of all targeted searches was determined to be 100%.

We decided against the testing of these more focused search approaches in our MEDLINE strategies (i.e., on their own) while keeping the high recall Embase search strategies. Embase is known for more exhaustive indexing than MEDLINE; therefore, focusing the search in MEDLINE only would be unlikely to reduce the number of references. MEDLINE is considered the gold standard for biomedical database searching and therefore did not make sense for us to limit our searches in MEDLINE while keeping the Embase search as sensitive as possible.

Further, we did not determine the effect of limiting specific PICO (Patient, Intervention, Comparison, Outcome) elements to major thesaurus terms done by previous researchers [12]. At CADTH, they concluded that it was safe to limit intervention concepts to major terms, but that limiting population elements to major terms should be avoided. However, the reviews included in our research covered a wider range of topics, many of which cannot easily be translated into the PICO framework. In this research, the consequences of focusing all elements in a search strategy are smaller than those by CADTH. Therefore, it was unnecessary to limit specific elements only. We left certain elements unchanged such as study types and population characteristics (such as age, gender, or species) because these are check tags which are never assigned as major terms.

Another limitation of this research is that all search strategies were designed by one searcher working at one institute. Methods used at Erasmus MC are designed to create highly sensitive sets of search terms in both thesaurus terms and in titles and abstracts [17]. It is unclear whether our conclusions are generalizable to other settings and institutions since other information specialists and review authors may not have similar emphasis on titles and abstract terms.

It must be stressed that decisions to limit search strategies for SRs should be made with close consideration to local context and resources. If review teams aim to find every article relevant to their research questions, imposing search limits should be carefully tested. Focusing search strategies should only be considered in those instances when the number of references retrieved is well beyond project resources. If the available resources permit screening all retrieved references, and their aim is to retrieve all relevant references, then focusing search results is not recommended, due to the, albeit small, chance of missing relevant references.

Further, reviewing high numbers of references may not be cost prohibitive for some SR teams. When reviewers use methods to review titles and abstracts within EndNote, the median number of articles reviewed per hour can be as high as 300, which is three times that observed for other methods [18]. Also other dedicated tools such as DistillerSR, Covidence, or Rayyan show promising results in speeding up the review process. When reviewers aim to reduce the time needed to screen titles and abstracts, changing methods used to screen titles and abstract, rather than limiting the recall of searches, may be a suitable alternative. Changing screening methods can reduce the total amount of time needed than focusing the searches and is less likely to result in relevant papers being missed.

Before focusing searches to obtain greater precision, SR searchers should remember that the number of relevant references retrieved is highly dependent on the selection of terms used to search titles and/or abstract fields. When limiting database searches to major terms, we recommend using highly sensitive search strategies that contain sufficient words and phrases in titles and/or abstracts. The methods used to develop search strategies that were used for these SRs are described in detail in a separate report [17]. This report describes a method developed by Erasmus MC librarians to help optimize the terms in the title and/or abstract fields. Because of the unique methods used to construct our searches, our conclusions should be viewed with caution, and we invite other information specialists to review their own data in a manner similar to our own.

## Conclusion

Systematic review searching aims to be optimally sensitive, but the resulting size of the biomedical literature retrieved often challenges the review teams to perform thorough screening. The question we aimed to answer is as follows: can researchers use focused searches to reduce the time burden of the screening process? If the number of search results retrieved is too high for the time and resource reviewers can dedicate to the screening process, search strategies in Embase alone or in both Embase and MEDLINE can be focused by searching for thesaurus terms as major descriptors. This approach may not ultimately have negative consequences in SRs, as long as a thorough search in other databases such as Web of Science next to the search in Embase and MEDLINE is performed. However, the reduction in the number of search results will in all likelihood be limited. Searching Embase and MEDLINE using terms in titles and abstracts alone results in too many relevant articles being missed and is therefore not recommended.

## Additional files

Additional file 1: Supplementary material. (XLSX 13 kb)

Additional file 2: STROBE Statement—Checklist of items that should be included in reports of cross-sectional studies. (DOCX 87 kb)

## Abbreviations

CADTH: Canadian Agency for Drugs and Technologies in Health
PICO: Patient, Intervention, Comparison, Outcome
RCTs: Randomized controlled trials
SRs: Systematic reviews
