# Supplementary material for: Searching Embase and MEDLINE by using only major descriptors or title and abstract fields: a prospective exploratory study
Source: Syst Rev. 2018 Nov 20;7:200. doi: 10.1186/s13643-018-0864-9 (PMC6247690; doi:10.1186/s13643-018-0864-9)
Supplement: Supplementary file 2 — STROBE Statement—Checklist of items that should be included in reports of cross-sectional studies. (DOCX 87 kb) [file 13643_2018_864_MOESM2_ESM.docx]

Supplementary material

**Table 1 : Example of adapted search strategies**

| **Embase.com** |
| --- |
| **original search strategy** |
| ('tennis elbow'/de OR ((elbow/de OR 'elbow disease'/exp OR 'elbow injury'/de OR (elbow*):ab,ti) AND ('tendinitis'/de OR 'tendon injury'/exp OR (tendinos* OR tendinit* OR tendinopath* OR ((tendon*) NEAR/3 (injur* OR trauma*))):ab,ti)) OR ((tennis NEXT/1 (elbow* OR arm*)) OR ((epicondyl* OR bursitis) NEAR/3 (humer* OR lateral* OR radiohumer*)) OR (chronic* NEAR/3 tendinopath*)):ab,ti) AND ('thrombocyte rich plasma'/de OR ((plasma/de) AND (injection/de)) OR 'blood autotransfusion'/de OR (((thrombocyte* OR platelet*) NEAR/3 (plasma)) OR PRP OR autotransfus* OR (autologous NEAR/3 blood)):ab,ti) |
| **search strategy major terms** |
| ('tennis elbow'/mj/de OR ((elbow/mj/de OR 'elbow disease'/mj/exp OR 'elbow injury'/mj/de OR (elbow*):ab,ti) AND ('tendinitis'/mj/de OR 'tendon injury'/mj/exp OR (tendinos* OR tendinit* OR tendinopath* OR ((tendon*) NEAR/3 (injur* OR trauma*))):ab,ti)) OR ((tennis NEXT/1 (elbow* OR arm*)) OR ((epicondyl* OR bursitis) NEAR/3 (humer* OR lateral* OR radiohumer*)) OR (chronic* NEAR/3 tendinopath*)):ab,ti) AND ('thrombocyte rich plasma'/mj/de OR ((plasma/mj/de) AND (injection/mj/de)) OR 'blood autotransfusion'/mj/de OR (((thrombocyte* OR platelet*) NEAR/3 (plasma)) OR PRP OR autotransfus* OR (autologous NEAR/3 blood)):ab,ti) |
| **search strategy title abstract** |
| ((elbow*):ab,ti) AND ((tendinos* OR tendinit* OR tendinopath* OR ((tendon*) NEAR/3 (injur* OR trauma*))):ab,ti)) OR ((tennis NEXT/1 (elbow* OR arm*)) OR ((epicondyl* OR bursitis) NEAR/3 (humer* OR lateral* OR radiohumer*)) OR (chronic* NEAR/3 tendinopath*)):ab,ti) AND ((((thrombocyte* OR platelet*) NEAR/3 (plasma)) OR PRP OR autotransfus* OR (autologous NEAR/3 blood)):ab,ti) |
| **Medline OvidSP** |
| **original search strategy** |
| ("Tennis Elbow"/ OR ((Elbow/ OR Elbow Joint/ OR (elbow*).ab,ti.) AND (exp "Tendon Injuries"/ OR (tendinos* OR tendinit* OR tendinopath* OR ((tendon*) ADJ3 (injur* OR trauma*))).ab,ti.)) OR ((tennis ADJ (elbow* OR arm*)) OR ((epicondyl* OR bursitis) ADJ3 (humer* OR lateral* OR radiohumer*)) OR (chronic* ADJ3 tendinopath*)).ab,ti.) AND ("Platelet-Rich Plasma"/ OR ((Plasma/) AND (exp injections/)) OR "Blood Transfusion, Autologous"/ OR (((thrombocyte* OR platelet*) ADJ3 (plasma)) OR PRP OR autotransfus* OR (autologous ADJ3 blood)).ab,ti.) |
| **search strategy major terms** |
| (*"Tennis Elbow"/ OR ((*Elbow/ OR *Elbow Joint/ OR (elbow*).ab,ti.) AND (exp *"Tendon Injuries"/ OR (tendinos* OR tendinit* OR tendinopath* OR ((tendon*) ADJ3 (injur* OR trauma*))).ab,ti.)) OR ((tennis ADJ (elbow* OR arm*)) OR ((epicondyl* OR bursitis) ADJ3 (humer* OR lateral* OR radiohumer*)) OR (chronic* ADJ3 tendinopath*)).ab,ti.) AND (*"Platelet-Rich Plasma"/ OR ((*Plasma/) AND (exp *injections/)) OR *"Blood Transfusion, Autologous"/ OR (((thrombocyte* OR platelet*) ADJ3 (plasma)) OR PRP OR autotransfus* OR (autologous ADJ3 blood)).ab,ti.) |
| **search strategy title abstract** |
| ((((elbow*).ab,ti.) AND ((tendinos* OR tendinit* OR tendinopath* OR ((tendon*) ADJ3 (injur* OR trauma*))).ab,ti.)) OR ((tennis ADJ (elbow* OR arm*)) OR ((epicondyl* OR bursitis) ADJ3 (humer* OR lateral* OR radiohumer*)) OR (chronic* ADJ3 tendinopath*)).ab,ti.) AND ((((thrombocyte* OR platelet*) ADJ3 (plasma)) OR PRP OR autotransfus* OR (autologous ADJ3 blood)).ab,ti.) |

**Table 2: Overview of articles included in this research**

|  | includes | unique references embase | unique references embase & medline | missed embase major | missed embase tiab | missed medline major | missed medline tiab |
| --- | --- | --- | --- | --- | --- | --- | --- |
| Adank, 2014 (1) | 4 | 0 | 0 | 0 | 0 | 0 | 0 |
| Ahmadi, 2015 (2) | 115 | 22 | 31 | 0 | 0 | 1 | 2 |
| Ambagtsheer, 2016 (3) | 5 | 0 | 0 | 0 | 0 | 0 | 0 |
| Arabkhani, 2015 (4) | 31 | 0 | 3 | 0 | 0 | 0 | 0 |
| Atiq, 2015 (5) | 11 | 2 | 2 | 0 | 0 | 0 | 0 |
| Baas, 2015 (6) | 14 | 0 | 0 | 0 | 0 | 0 | 0 |
| Balak, 2016 (7) | 92 | 4 | 9 | 3 | 3 | 2 | 2 |
| Bijlard, 2015 (8) | 18 | 0 | 0 | 0 | 0 | 0 | 0 |
| Blokker, 2016 (9) | 16 | 1 | 2 | 0 | 1 | 0 | 0 |
| Boersema, 2016 (10) | 48 | 3 | 11 | 0 | 1 | 1 | 1 |
| Caron, 2015 (11) | 8 | 1 | 1 | 0 | 0 | 0 | 0 |
| Caron, 2015 (12) | 16 | 0 | 1 | 0 | 0 | 0 | 1 |
| Claessen, 2014 (13) | 31 | 2 | 8 | 1 | 2 | 3 | 4 |
| Claessen, 2015 (14) | 22 | 0 | 0 | 0 | 0 | 0 | 0 |
| Cnossen, 2016 (15) | 25 | 0 | 0 | 0 | 0 | 0 | 0 |
| De Bruijn, 2015 (16) | 26 | 0 | 0 | 0 | 0 | 0 | 0 |
| de Jong, 2016 (17) | 53 | 5 | 15 | 1 | 5 | 0 | 6 |
| De Lima, 2015 (18) | 77 | 11 | 26 | 9 | 9 | 2 | 5 |
| de Vos, 2014 (19) | 7 | 0 | 0 | 0 | 0 | 0 | 0 |
| de Vos-Kerkhof, 2016 (20) | 57 | 10 | 17 | 3 | 3 | 2 | 3 |
| de Vries, 2015 (21) | 14 | 0 | 0 | 0 | 0 | 0 | 0 |
| Ekkelenkamp, 2016 (22) | 94 | 3 | 16 | 0 | 2 | 1 | 12 |
| Fischer, 2014 (23) | 101 | 6 | 15 | 0 | 2 | 5 | 5 |
| Garcia, 2016 (24) | 24 | 2 | 6 | 2 | 2 | 1 | 4 |
| Hakim, 2017 (25) | 80 | 12 | 16 | 2 | 2 | 0 | 0 |
| Hanewinckel, 2016 (26) | 58 | 6 | 18 | 0 | 0 | 1 | 1 |
| Harrison, 2015 (27) | 15 | 0 | 0 | 0 | 0 | 0 | 0 |
| Hassing, 2015 (28) | 11 | 0 | 0 | 0 | 0 | 0 | 0 |
| Hogendoorn, 2014 (29) | 47 | 3 | 4 | 2 | 3 | 1 | 1 |
| Hosnijeh, 2015 (30) | 25 | 6 | 7 | 0 | 0 | 0 | 0 |
| Jaspers, 2016 (31) | 90 | 2 | 4 | 0 | 0 | 2 | 2 |
| Kortram, 2014 (32) | 21 | 3 | 8 | 2 | 2 | 1 | 3 |
| Kragt, 2016 (33) | 90 | 2 | 4 | 1 | 1 | 0 | 0 |
| Kroese, 2016 (34) | 13 | 0 | 0 | 0 | 0 | 0 | 0 |
| Kuhlmann, 2016 (35) | 41 | 2 | 6 | 0 | 0 | 1 | 3 |
| Lafranca, 2015 (36) | 57 | 3 | 5 | 0 | 0 | 1 | 1 |
| Leermakers, 2015 (37) | 50 | 11 | 28 | 1 | 11 | 2 | 3 |
| Lenk, 2016 (38) | 24 | 2 | 4 | 0 | 1 | 0 | 2 |
| Li, 2015 (39) | 13 | 1 | 1 | 0 | 0 | 0 | 0 |
| Liebregts, 2015 (40) | 24 | 0 | 2 | 0 | 0 | 0 | 0 |
| Ligthart, 2017 (41) | 10 | 0 | 0 | 0 | 0 | 0 | 0 |
| Lijster, 2017 (42) | 20 | 0 | 1 | 0 | 0 | 0 | 0 |
| Luk, 2015 (43) | 62 | 13 | 17 | 5 | 13 | 0 | 1 |
| Mookhoek, 2016 (44) | 60 | 4 | 14 | 0 | 4 | 0 | 1 |
| Mosler, 2015 (45) | 17 | 0 | 0 | 0 | 0 | 0 | 0 |
| Muka, 2016 (46) | 10 | 1 | 1 | 0 | 0 | 0 | 0 |
| Muka, 2016 (47) | 85 | 2 | 7 | 0 | 0 | 3 | 3 |
| Muka, 2016 (48) | 59 | 1 | 1 | 0 | 0 | 0 | 0 |
| Nano, 2016 (49) | 43 | 1 | 5 | 0 | 0 | 0 | 4 |
| Plomp, 2016 (50) | 29 | 1 | 1 | 1 | 1 | 0 | 0 |
| Rodenburg-Vlot, 2016 (51) | 46 | 0 | 2 | 0 | 0 | 0 | 0 |
| Roelants, 2016 (52) | 17 | 1 | 2 | 1 | 1 | 1 | 1 |
| Rokx, 2015 (53) | 19 | 0 | 1 | 0 | 0 | 0 | 0 |
| Schoots, 2015 (54) | 16 | 0 | 0 | 0 | 0 | 0 | 0 |
| Serner, 2015 (55) | 72 | 0 | 1 | 0 | 0 | 0 | 0 |
| Strang, 2016 (56) | 16 | 0 | 0 | 0 | 0 | 0 | 0 |
| Suijkerbuijk, 2015 (57) | 18 | 0 | 1 | 0 | 0 | 0 | 0 |
| Swart, 2016 (58) | 31 | 1 | 1 | 1 | 1 | 0 | 0 |
| Taneri, 2016 (59) | 10 | 0 | 1 | 0 | 0 | 0 | 0 |
| Ten Kate, 2015 (60) | 14 | 0 | 0 | 0 | 0 | 0 | 0 |
| Tielemans, 2016 (61) | 40 | 0 | 1 | 0 | 0 | 0 | 0 |
| Tromp, 2016 (62) | 27 | 0 | 1 | 0 | 0 | 1 | 1 |
| van der Does, 2016 (63) | 11 | 0 | 0 | 0 | 0 | 0 | 0 |
| van der Valk, 2014 (64) | 16 | 1 | 1 | 0 | 0 | 0 | 0 |
| van Dijk, 2015 (65) | 4 | 0 | 0 | 0 | 0 | 0 | 0 |
| van Mol, 2015 (66) | 40 | 2 | 8 | 0 | 0 | 2 | 2 |
| van Waardhuizen, 2014 (67) | 13 | 0 | 0 | 0 | 0 | 0 | 0 |
| Vargas, 2016 (68) | 69 | 9 | 28 | 0 | 8 | 3 | 15 |
| Voogt, 2015 (69) | 14 | 0 | 0 | 0 | 0 | 0 | 0 |
| Wesseloo, 2016 (70) | 48 | 0 | 1 | 0 | 0 | 0 | 0 |
| Wu, 2016 (71) | 22 | 1 | 1 | 1 | 1 | 0 | 0 |
| Xavier Harmeling, 2015 (72) | 14 | 0 | 1 | 0 | 0 | 1 | 1 |
| Younge, 2015 (73) | 11 | 0 | 1 | 0 | 0 | 0 | 1 |

1. Adank MC, van Dorp W, Smit M, van Casteren NJ, Laven JS, Pieters R, et al. Electroejaculation as a method of fertility preservation in boys diagnosed with cancer: a single-center experience and review of the literature. Fertil Steril. 2014 Jul;102(1):199-205 e1.

2. Ahmadi AR, Lafranca JA, Claessens LA, Imamdi RM, JN IJ, Betjes MG, et al. Shifting paradigms in eligibility criteria for live kidney donation: a systematic review. Kidney Int. 2015 Jan;87(1):31-45.

3. Ambagtsheer F, de Jong J, Bramer WM, Weimar W. On Patients Who Purchase Organ Transplants Abroad. Am J Transplant. 2016 Oct;16(10):2800-15.

4. Arabkhani B, Mookhoek A, Di Centa I, Lansac E, Bekkers JA, De Lind Van Wijngaarden R, et al. Reported Outcome After Valve-Sparing Aortic Root Replacement for Aortic Root Aneurysm: A Systematic Review and Meta-Analysis. Ann Thorac Surg. 2015 Sep;100(3):1126-31.

5. Atiq F, van den Bemt PM, Leebeek FW, van Gelder T, Versmissen J. A systematic review on the accumulation of prophylactic dosages of low-molecular-weight heparins (LMWHs) in patients with renal insufficiency. Eur J Clin Pharmacol. 2015 Aug;71(8):921-9.

6. Baas M, Duraku LS, Corten EM, Mureau MA. A systematic review on the sensory reinnervation of free flaps for tongue reconstruction: Does improved sensibility imply functional benefits? J Plast Reconstr Aesthet Surg. 2015 Aug;68(8):1025-35.

7. Balak DM, Fallah Arani S, Hajdarbegovic E, Hagemans CA, Bramer WM, Thio HB, et al. Efficacy, effectiveness and safety of fumaric acid esters in the treatment of psoriasis: a systematic review of randomized and observational studies. Br J Dermatol. 2016 Aug;175(2):250-62.

8. Bijlard E, Steltenpool S, Niessen FB. Intralesional 5-fluorouracil in keloid treatment: a systematic review. Acta Derm Venereol. 2015 Sep;95(7):778-82.

9. Blokker BM, Wagensveld IM, Weustink AC, Oosterhuis JW, Hunink MG. Non-invasive or minimally invasive autopsy compared to conventional autopsy of suspected natural deaths in adults: a systematic review. Eur Radiol. 2016 Apr;26(4):1159-79.

10. Boersema GS, Grotenhuis N, Bayon Y, Lange JF, Bastiaansen-Jenniskens YM. The Effect of Biomaterials Used for Tissue Regeneration Purposes on Polarization of Macrophages. Biores Open Access. 2016;5(1):6-14.

11. Caron CJ, Pluijmers BI, Joosten KF, Mathijssen IM, van der Schroeff MP, Dunaway DJ, et al. Feeding difficulties in craniofacial microsomia: a systematic review. Int J Oral Maxillofac Surg. 2015 Jun;44(6):732-7.

12. Caron CJ, Pluijmers BI, Joosten KF, Mathijssen IM, van der Schroeff MP, Dunaway DJ, et al. Obstructive sleep apnoea in craniofacial microsomia: a systematic review. Int J Oral Maxillofac Surg. 2015 May;44(5):592-8.

13. Claessen FM, de Vos RJ, Reijman M, Meuffels DE. Predictors of primary Achilles tendon ruptures. Sports Med. 2014 Sep;44(9):1241-59.

14. Claessen FM, Louwerens JK, Doornberg JN, van Dijk CN, van den Bekerom MP, Eygendaal D. Hegemann's disease and fishtail deformity: aetiopathogenesis, radiographic appearance and clinical outcome. J Child Orthop. 2015 Feb;9(1):1-8.

15. Cnossen MC, Scholten AC, Lingsma HF, Synnot A, Tavender E, Gantner D, et al. Adherence to Guidelines in Adult Patients with Traumatic Brain Injury: A Living Systematic Review. J Neurotrauma. 2016 Aug 25.

16. De Bruijn KM, van Eijck CH. New-onset diabetes after distal pancreatectomy: a systematic review. Ann Surg. 2015 May;261(5):854-61.

17. de Jong MH, Kamperman AM, Oorschot M, Priebe S, Bramer W, van de Sande R, et al. Interventions to Reduce Compulsory Psychiatric Admissions: A Systematic Review and Meta-analysis. JAMA Psychiatry. 2016 Jul 1;73(7):657-64.

18. De Lima A, Galjart B, Wisse PH, Bramer WM, van der Woude CJ. Does lower gastrointestinal endoscopy during pregnancy pose a risk for mother and child? - a systematic review. BMC Gastroenterol. 2015 Feb 12;15:15.

19. de Vos RJ, Windt J, Weir A. Strong evidence against platelet-rich plasma injections for chronic lateral epicondylar tendinopathy: a systematic review. Br J Sports Med. 2014 Jun;48(12):952-6.

20. de Vos-Kerkhof E, Geurts DH, Wiggers M, Moll HA, Oostenbrink R. Tools for 'safety netting' in common paediatric illnesses: a systematic review in emergency care. Arch Dis Child. 2016 Feb;101(2):131-9.

21. de Vries J, Ischebeck BK, Voogt LP, van der Geest JN, Janssen M, Frens MA, et al. Joint position sense error in people with neck pain: A systematic review. Man Ther. 2015 Dec;20(6):736-44.

22. Ekkelenkamp VE, Koch AD, de Man RA, Kuipers EJ. Training and competence assessment in GI endoscopy: a systematic review. Gut. 2016 Apr;65(4):607-15.

23. Fischer C, Lingsma HF, Marang-van de Mheen PJ, Kringos DS, Klazinga NS, Steyerberg EW. Is the readmission rate a valid quality indicator? A review of the evidence. PLoS One. 2014;9(11):e112282.

24. Garcia AH, Voortman T, Baena CP, Chowdhurry R, Muka T, Jaspers L, et al. Maternal weight status, diet, and supplement use as determinants of breastfeeding and complementary feeding: a systematic review and meta-analysis. Nutr Rev. 2016 Aug;74(8):490-516.

25. Hakim MS, Wang W, Bramer WM, Geng J, Huang F, de Man RA, et al. The global burden of hepatitis E outbreaks: a systematic review. Liver Int. 2017 Jan;37(1):19-31.

26. Hanewinckel R, van Oijen M, Ikram MA, van Doorn PA. The epidemiology and risk factors of chronic polyneuropathy. Eur J Epidemiol. 2016 Jan;31(1):5-20.

27. Harrison SL, Sajjad A, Bramer WM, Ikram MA, Tiemeier H, Stephan BC. Exploring strategies to operationalize cognitive reserve: A systematic review of reviews. J Clin Exp Neuropsychol. 2015;37(3):253-64.

28. Hassing RJ, Alsma J, Arcilla MS, van Genderen PJ, Stricker BH, Verbon A. International travel and acquisition of multidrug-resistant Enterobacteriaceae: a systematic review. Euro Surveill. 2015;20(47).

29. Hogendoorn W, Lavida A, Hunink MG, Moll FL, Geroulakos G, Muhs BE, et al. Open repair, endovascular repair, and conservative management of true splenic artery aneurysms. J Vasc Surg. 2014 Dec;60(6):1667-76 e1.

30. Hosnijeh FS, Runhaar J, van Meurs JB, Bierma-Zeinstra SM. Biomarkers for osteoarthritis: Can they be used for risk assessment? A systematic review. Maturitas. 2015 Sep;82(1):36-49.

31. Jaspers L, Feys F, Bramer WM, Franco OH, Leusink P, Laan ET. Efficacy and Safety of Flibanserin for the Treatment of Hypoactive Sexual Desire Disorder in Women: A Systematic Review and Meta-analysis. JAMA Intern Med. 2016 Apr;176(4):453-62.

32. Kortram K, Lafranca JA, JN IJ, Dor FJ. The need for a standardized informed consent procedure in live donor nephrectomy: a systematic review. Transplantation. 2014 Dec 15;98(11):1134-43.

33. Kragt L, Dhamo B, Wolvius EB, Ongkosuwito EM. The impact of malocclusions on oral health-related quality of life in children-a systematic review and meta-analysis. Clin Oral Investig. 2016 Nov;20(8):1881-94.

34. Kroese LF, de Smet GH, Jeekel J, Kleinrensink GJ, Lange JF. Systematic Review and Meta-Analysis of Extraperitoneal Versus Transperitoneal Colostomy for Preventing Parastomal Hernia. Dis Colon Rectum. 2016 Jul;59(7):688-95.

35. Kuhlmann AY, Etnel JR, Roos-Hesselink JW, Jeekel J, Bogers AJ, Takkenberg JJ. Systematic review and meta-analysis of music interventions in hypertension treatment: a quest for answers. BMC Cardiovasc Disord. 2016 Apr 19;16:69.

36. Lafranca JA, JN IJ, Betjes MG, Dor FJ. Body mass index and outcome in renal transplant recipients: a systematic review and meta-analysis. BMC Med. 2015 May 12;13:111.

37. Leermakers ET, Moreira EM, Kiefte-de Jong JC, Darweesh SK, Visser T, Voortman T, et al. Effects of choline on health across the life course: a systematic review. Nutr Rev. 2015 Aug;73(8):500-22.

38. Lenk EJ, Redekop WK, Luyendijk M, Rijnsburger AJ, Severens JL. Productivity Loss Related to Neglected Tropical Diseases Eligible for Preventive Chemotherapy: A Systematic Literature Review. PLoS Negl Trop Dis. 2016 Feb;10(2):e0004397.

39. Li J, Hernanda PY, Bramer WM, Peppelenbosch MP, van Luijk J, Pan Q. Anti-tumor effects of metformin in animal models of hepatocellular carcinoma: a systematic review and meta-analysis. PLoS One. 2015;10(6):e0127967.

40. Liebregts M, Vriesendorp PA, Mahmoodi BK, Schinkel AF, Michels M, ten Berg JM. A Systematic Review and Meta-Analysis of Long-Term Outcomes After Septal Reduction Therapy in Patients With Hypertrophic Cardiomyopathy. JACC Heart Fail. 2015 Nov;3(11):896-905.

41. Ligthart KAM, Buitendijk L, Koes BW, van Middelkoop M. The association between ethnicity, socioeconomic status and compliance to pediatric weight-management interventions - A systematic review. Obes Res Clin Pract. 2017 Sep - Oct;11(5 Suppl 1):1-51.

42. Lijster JM, Dierckx B, Utens EM, Verhulst FC, Zieldorff C, Dieleman GC, et al. The Age of Onset of Anxiety Disorders. Can J Psychiatry. 2017 Apr;62(4):237-46.

43. Luk F, de Witte SF, Bramer WM, Baan CC, Hoogduijn MJ. Efficacy of immunotherapy with mesenchymal stem cells in man: a systematic review. Expert Rev Clin Immunol. 2015 May;11(5):617-36.

44. Mookhoek A, Korteland NM, Arabkhani B, Di Centa I, Lansac E, Bekkers JA, et al. Bentall Procedure: A Systematic Review and Meta-Analysis. Ann Thorac Surg. 2016 May;101(5):1684-9.

45. Mosler AB, Agricola R, Weir A, Holmich P, Crossley KM. Which factors differentiate athletes with hip/groin pain from those without? A systematic review with meta-analysis. Br J Sports Med. 2015 Jun;49(12):810.

46. Muka T, Nano J, Voortman T, Braun KVE, Ligthart S, Stranges S, et al. The role of global and regional DNA methylation and histone modifications in glycemic traits and type 2 diabetes: A systematic review. Nutr Metab Cardiovasc Dis. 2016 Jul;26(7):553-66.

47. Muka T, Koromani F, Portilla E, O'Connor A, Bramer WM, Troup J, et al. The role of epigenetic modifications in cardiovascular disease: A systematic review. Int J Cardiol. 2016 Jun 1;212:174-83.

48. Muka T, Vargas KG, Jaspers L, Wen KX, Dhana K, Vitezova A, et al. Estrogen receptor beta actions in the female cardiovascular system: A systematic review of animal and human studies. Maturitas. 2016 Apr;86:28-43.

49. Nano J, Muka T, Cepeda M, Voortman T, Dhana K, Brahimaj A, et al. Association of circulating total bilirubin with the metabolic syndrome and type 2 diabetes: A systematic review and meta-analysis of observational evidence. Diabetes Metab. 2016 Dec;42(6):389-97.

50. Plomp RG, van Lieshout MJ, Joosten KF, Wolvius EB, van der Schroeff MP, Versnel SL, et al. Treacher Collins Syndrome: A Systematic Review of Evidence-Based Treatment and Recommendations. Plast Reconstr Surg. 2016 Jan;137(1):191-204.

51. Rodenburg-Vlot MB, Ruytjens L, Oostenbrink R, Goedegebure A, van der Schroeff MP. Systematic Review: Incidence and Course of Hearing Loss Caused by Bacterial Meningitis: In Search of an Optimal Timed Audiological Follow-up. Otol Neurotol. 2016 Jan;37(1):1-8.

52. Roelants JA, de Jonge RC, Steegers-Theunissen RP, Reiss IK, Joosten KF, Vermeulen MJ. Prenatal markers of neonatal fat mass: A systematic review. Clin Nutr. 2016 Oct;35(5):995-1007.

53. Rokx C, Rijnders BJ, van Laar JA. Treatment of multicentric Castleman&rsquo;s disease in HIV-1 infected and uninfected patients: a systematic review. Neth J Med. 2015 Jun;73(5):202-10.

54. Schoots IG, Roobol MJ, Nieboer D, Bangma CH, Steyerberg EW, Hunink MG. Magnetic resonance imaging-targeted biopsy may enhance the diagnostic accuracy of significant prostate cancer detection compared to standard transrectal ultrasound-guided biopsy: a systematic review and meta-analysis. Eur Urol. 2015 Sep;68(3):438-50.

55. Serner A, van Eijck CH, Beumer BR, Holmich P, Weir A, de Vos RJ. Study quality on groin injury management remains low: a systematic review on treatment of groin pain in athletes. Br J Sports Med. 2015 Jun;49(12):813.

56. Strang SG, Van Lieshout EM, Van Waes OJ, Verhofstad MH. Prevalence and mortality of abdominal compartment syndrome in severely injured patients: A systematic review. J Trauma Acute Care Surg. 2016 Sep;81(3):585-92.

57. Suijkerbuijk MAM, Reijman M, Lodewijks SJ, Punt J, Meuffels DE. Hamstring Tendon Regeneration After Harvesting: A Systematic Review. Am J Sports Med. 2015 Oct;43(10):2591-8.

58. Swart NM, van Oudenaarde K, Reijnierse M, Nelissen RG, Verhaar JA, Bierma-Zeinstra SM, et al. Effectiveness of exercise therapy for meniscal lesions in adults: A systematic review and meta-analysis. J Sci Med Sport. 2016 Dec;19(12):990-8.

59. Taneri PE, Kiefte-de Jong JC, Bramer WM, Daan NM, Franco OH, Muka T. Association of alcohol consumption with the onset of natural menopause: a systematic review and meta-analysis. Hum Reprod Update. 2016 Jun;22(4):516-28.

60. Ten Kate CA, Tibboel D, Kraemer US. B-type natriuretic peptide as a parameter for pulmonary hypertension in children. A systematic review. Eur J Pediatr. 2015 Oct;174(10):1267-75.

61. Tielemans MJ, Garcia AH, Peralta Santos A, Bramer WM, Luksa N, Luvizotto MJ, et al. Macronutrient composition and gestational weight gain: a systematic review. Am J Clin Nutr. 2016 Jan;103(1):83-99.

62. Tromp K, Zwaan CM, van de Vathorst S. Motivations of children and their parents to participate in drug research: a systematic review. Eur J Pediatr. 2016 May;175(5):599-612.

63. van der Does Y, Rood PP, Haagsma JA, Patka P, van Gorp EC, Limper M. Procalcitonin-guided therapy for the initiation of antibiotics in the ED: a systematic review. Am J Emerg Med. 2016 Jul;34(7):1286-93.

64. van der Valk JP, Dubois AE, Gerth van Wijk R, Wichers HJ, de Jong NW. Systematic review on cashew nut allergy. Allergy. 2014 Jun;69(6):692-8.

65. van Dijk GM, Maneva M, Colpani V, Dhana K, Muka T, Jaspers L, et al. The association between vasomotor symptoms and metabolic health in peri- and postmenopausal women: a systematic review. Maturitas. 2015 Feb;80(2):140-7.

66. van Mol MM, Kompanje EJ, Benoit DD, Bakker J, Nijkamp MD. The Prevalence of Compassion Fatigue and Burnout among Healthcare Professionals in Intensive Care Units: A Systematic Review. PLoS One. 2015;10(8):e0136955.

67. van Waardhuizen CN, Langhout M, Ly F, Braun L, Genders TS, Petersen SE, et al. Diagnostic performance and comparative cost-effectiveness of non-invasive imaging tests in patients presenting with chronic stable chest pain with suspected coronary artery disease: a systematic overview. Curr Cardiol Rep. 2014;16(10):537.

68. Vargas KG, Milic J, Zaciragic A, Wen KX, Jaspers L, Nano J, et al. The functions of estrogen receptor beta in the female brain: A systematic review. Maturitas. 2016 Nov;93:41-57.

69. Voogt L, de Vries J, Meeus M, Struyf F, Meuffels D, Nijs J. Analgesic effects of manual therapy in patients with musculoskeletal pain: a systematic review. Man Ther. 2015 Apr;20(2):250-6.

70. Wesseloo R, Kamperman AM, Munk-Olsen T, Pop VJ, Kushner SA, Bergink V. Risk of Postpartum Relapse in Bipolar Disorder and Postpartum Psychosis: A Systematic Review and Meta-Analysis. Am J Psychiatry. 2016 Feb 1;173(2):117-27.

71. Wu Z, van de Haar RC, Sparreboom CL, Boersema GS, Li Z, Ji J, et al. Is the intraoperative air leak test effective in the prevention of colorectal anastomotic leakage? A systematic review and meta-analysis. Int J Colorectal Dis. 2016 Aug;31(8):1409-17.

72. Xavier Harmeling J, Kouwenberg CA, Bijlard E, Burger KN, Jager A, Mureau MA. The effect of immediate breast reconstruction on the timing of adjuvant chemotherapy: a systematic review. Breast Cancer Res Treat. 2015 Sep;153(2):241-51.

73. Younge JO, Gotink RA, Baena CP, Roos-Hesselink JW, Hunink MG. Mind-body practices for patients with cardiac disease: a systematic review and meta-analysis. Eur J Prev Cardiol. 2015 Nov;22(11):1385-98.
